
JOURNAL INFORMATION
==============================
NLM Title Abbreviation: Behav Neurol
Journal ID: Behav Neurol
NLM Journal ID: 8914585
Journal ID: BN

Behavioural Neurology

ISSN: 0953-4180
EISSN: 1875-8584
Publisher: Wiley

ARTICLE INFORMATION
==============================
PMCID: PMC5434406
PMID: 21422551
DOI: 10.3233/BEN-2010-0291
Article ID: 149360
Article version: 1
Subjects: Research Article

Absence of Right Visual Field Attentional Bias in Reading in Dyslexic Children

Bricout-Tomasi Laure
Billard Catherine
Siéroff Eric *
Laboratoire de Psychologie et Neuropsychologie Cognitives (FRE 3292) Institut de Psychologie Paris Descartes University Boulogne-Billancourt France
*Eric Siéroff: eric.sieroff@parisdescartes.fr
Print publication date: 2010
Electronic publication date: 2011 Mar 7
Volume: 23
Issue: 4
First page: 189
Last page: 191
Received 2011 Jan 31; Accepted 2011 Jan 31
Copyright: Copyright © 2010 Hindawi Publishing Corporation and the authors.
Copyright year: 2010
License: This is an open access article distributed under the Creative Commons Attribution License, which permits unrestricted use, distribution, and reproduction in any medium, provided the original work is properly cited.
License URL: https://creativecommons.org/licenses/by/3.0/

==============================
Behavioural Neurology 23 (2010) 189–191
DOI 10.3233/BEN-2010-0291
IOS Press

### Absence of right visual field attentional bias in reading in dyslexic children

Laure Bricout-Tomasi, Catherine Billard and Eric Siéroff*
*Laboratoire de Psychologie et Neuropsychologie Cognitives* (*FRE 3292*), *Institut de Psychologie, Paris Descartes University, Boulogne-Billancourt, France*

### 1. Introduction

A parafoveal word is better identified in the right visual field (RVF) than in the left visual field (LVF), in adults [4] and in school-age children [5]. Attention may play a role, at least partially, in this RVF superiority in reading. The hypothesis is that attention is not symmetrically distributed in the state of expectancy prior to the presentation of parafoveal words, and a bias in favor of the RVF exists. This attentional bias may be the consequence of the left hemispheric control when the subject is engaged in a linguistic task [2], and of scanning habits [1]. Many investigators have hypothesized a failure of left-hemisphere posterior brain systems to function properly during reading in dyslexia [6]. A possibility is that this hemispheric dysfunctioning may influence the distribution of spatial attention and affect the RVF bias in reading.

We studied the distribution of attention when identifying parafoveal words in dyslexic children and age-matched skilled readers. We compared performance in a condition in which a word was presented alone in the LVF or RVF, and a condition in which a distractor was simultaneously presented in the visual field opposite the word. The distribution of attention should be affected by the presence of the distractor, and the distractor effect is stronger in the LVF than in the RVF in normal adult readers [7]. If dyslexic children have difficulties controlling the distribution of attention in reading, they should demonstrate a greater distractor effect than skilled readers of same age, specifically in the RVF. We also presented a bilateral condition in which two words, one in each visual field, had to be identified. If the difference between dyslexics and skilled readers mainly concerns the capacity to identify and report visual words, the main difference should occur in the bilateral condition.

### 2. Methods

40 dyslexic children (mean age $= 11.4$, $\sigma = 9.3$) and 40 controls (mean age $= 11.0$, $\sigma = 5.8$) participated to the experiment. Dyslexic children had a reading delay of at least 24 months on the Alouette Reading Test [3]. None had a history of neurological illness.

120 four-letter words (width of 1.5° of visual angle) were presented in the LVF or the RVF, with an eccentricity of 1.4° between the fixation item (a cross) and the nearest extremity. Words were presented unilaterally (one word), alone or accompanied with a distractor made of 4 xs in the opposite visual field, or bilaterally (two words). The presentation duration of words was adapted to each participant in order to obtain about 40–70% of correct identification. A patterned mask immediately followed the word. Participants were required to fixate the cross, and to identify and orally report the word(s).

*Corresponding author: Eric Siéroff, Laboratoire de Psychologie et Neuropsychologie Cognitives (FRE 3292), Institut de Psychologie, Paris Descartes University, 71, avenue Edouard Vaillant, 92100 Boulogne-Billancourt, France. E-mail: eric.sieroff@parisdescartes.fr.

ISSN 0953-4180/10/$27.50 © 2010 – IOS Press and the authors. All rights reserved

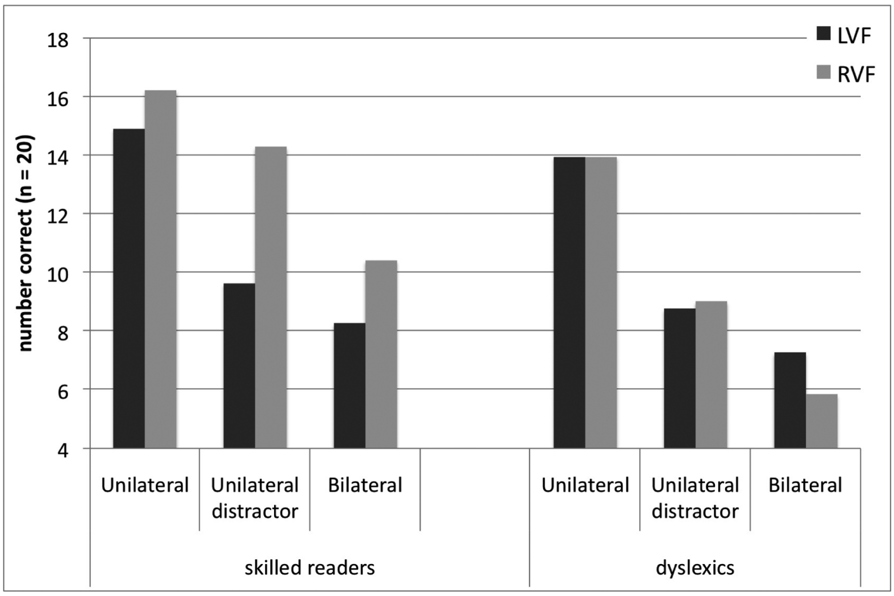

Fig. 1. Number of correctly identified words in the LVF and RVF in dyslexic and skilled reader children.

### 3. Results

Unsurprisingly, the mean presentation duration was much superior in dyslexic children (339 ms) than in skilled readers (162 ms) in order to obtain comparable identification rates.

An ANOVA was conducted on the number of correct responses ($n$ = 20), with Reading level (dyslexic children, skilled readers) as between-factor, and Presentation type (unilateral, unilateral with distractor, bilateral) and Visual field (LVF, RVF) as within factors.

Performance was the best in the unilateral condition ($m$ = 14.8) compared to the unilateral with distractor (10.4), and the bilateral conditions (7.9). The interaction Reading level x Presentation type, F(2,152) = 4.03; $p < 0.05$, showed that dyslexics were affected by the presence of a second stimulus (distractor or word) more than skilled readers. Most importantly, the difference between the unilateral and the unilateral with distractor conditions was larger in dyslexics ($m$ = 5.07) than in skilled readers (3.60), showing difficulties to ignore the distractor in dyslexics, but the difference between the unilateral with distractor and the bilateral conditions was equivalent in both groups (2.34 and 2.63, respectively).

The interaction Reading level x Visual field was also significant, F(2,76) = 8.91; $p < 0.001$. The dyslexic children did not show the global RVF superiority, contrarily to the skilled readers. Also, skilled readers showed a stronger effect of a second stimulus (distractor or word) in the LVF than in the RVF, but dyslexics did not show this visual field difference and had the same effect of a second stimulus in both visual fields (see Fig. 1).

Results (particularly the absence of asymmetry in dyslexics) were confirmed in a second experiment reducing the presentation duration (maximum 200 ms) in a new group of 20 dyslexics with similar characteristics, thus may not be attributed to the different presentation duration between groups, and to possible saccades during the trials in dyslexics.

### 4. Discussion

Dyslexics needed presentation duration twice as much as the skilled readers to obtain similar reading performance, showing a strong difficulty to identify parafoveal words. However, when similar performance was obtained between both groups, several differences were still found. First, dyslexics were more affected by the presence of a second stimulus (a distractor as well as a second word) than skilled readers. Thus, the main change between our skilled readers and dyslexics may not reside in the capacity to report several words, but in the ability to distribute spatial attentional resources in reading.

Second, although skilled readers showed a strong superiority of RVF in reading, no visual field asymmetry was found in dyslexics. Moreover, only skilled readers showed a stronger effect of a second stimulus in the LVF than in the RVF. Apparently, dyslexics may read as if they had two LVFs. These results suggest that skilled readers presented an attentional bias in favor of the RVF, and dyslexics may distribute attention more equally between visual fields. This absence of RVF bias in dyslexics may be the result of a left hemisphere dysfunctioning.

### References

[1] W. Heron, Perception as a function of retinal locus and attention, *The American Journal of Psychology* **70** (1957), 38–48.

[2] K.M. Kinsbourne, The cerebral basis of lateral asymmetries in attention, *Acta Psychologica* **33** (1970), 193–201.

[3] P. Lefavrais, *Test de l'Alouette*, Editions du centre de psychologie appliquée, Paris, 1965.

[4] M. Mishkin and D.G. Forgays, Word recognition as a function of retinal locus, *Journal of Experimental Psychology* **43** (1952), 43–48.

[5] M.E. Olson, Laterality differences in tachistoscopic word recognition in normal and delayed readers in elementary school, *Neuropsychologia* **11** (1973), 343–350.

[6] S.E. Shaywitz, M. Mody and B.A. Shaywitz, Neural mechanisms in dyslexia, *Current Directions in Psychological Science* **15** (2002), 278–281.

[7] E. Siéroff and M. Urbanski, Conditions of visual verbal extinction: Does the ipsilesional stimulus have to be identified? *Brain and Cognition* **48** (2002), 563–569.
